# W2A‐28: a novel benzamide‐based dual modulator of class I HDACs and Wnt/ß‐catenin signaling for the treatment of Alzheimer's disease

**DOI:** 10.1002/alz70859_100859

**Published:** 2025-12-25

**Authors:** Wenyan Lu, Thomas R. Caulfield, Suren Jeevaratnam, Guojun Bu, Takahisa Kanekiyo, Yonghe Li

**Affiliations:** ^1^ Mayo Clinic, Jacksonville, FL USA

## Abstract

**Background:**

Alzheimer’s disease (AD) is a multifactorial disease often with mixed pathologies. As such, drugs targeting multiple pathological processes simultaneously could have synergistic therapeutic effects. The histone acetylation homeostasis is greatly impaired in AD, and histone deacetylase (HDAC) inhibitors have been known to alleviate AD‐relevant pathologies in various animal models. In addition, Wnt/β‐catenin signaling is compromised in AD, and restoring Wnt/β‐catenin signaling is an attractive therapeutic strategy for AD treatment. CI‐994 is a class I HDAC inhibitor containing N‐(2‐aminophenyl)‐benzamide. Our recent studies indicate that CI‐994 is also an activator of Wnt/β‐catenin signaling by stabilizing Wnt co‐receptor LRP6.

**Method:**

We use CI‐994 as a scaffold to develop novel potent dual modulators for class I HDAC inhibition and Wnt/β‐catenin signaling activation, and then determine the therapeutic potential of the lead compound W2A‐28 in AD patient‐specific iPSC‐derived cerebral organoids.

**Result:**

W2A‐28 inhibits class I HDAC1, 2 and 3 activities with IC_50_ values of 512 nM, 675 nM and 217 nM, respectively, with no inhibitory activities on other HDACs and Sirtuin family members. Furthermore, W2A‐28 greatly increases Wnt reporter activity with an EC_50_ value of 1.61 µM in Wnt‐3A‐expressing HEK293 cells. As expected, activation of Wnt/β‐catenin signaling by W2A‐28 is associated with elevated Wnt co‐receptor LRP6 protein level by reducing LRP6 degradation. Importantly, W2A‐28 displays excellent aqueous solubility and microsomal stability. Further, W2A‐28 shows high permeability with no active efflux in MDR1‐MDCKII permeability assays and is not a P‐gp substrate. Finally, W2A‐28 significantly reduces Aβ40 and Aβ42 levels and suppresses tau phosphorylation in AD patient‐specific iPSC‐derived cerebral organoids carrying APOE4.

**Conclusion:**

Our studies suggest that W2A‐28 is a potential drug candidate for the treatment of AD.

